# Sex differences in the prefrontal cortex and muscle oxygenation during exercise until exhaustion in endurance‐trained individuals

**DOI:** 10.1113/EP093287

**Published:** 2025-11-29

**Authors:** Daniel Ramos‐López, Raúl Caulier‐Cisterna, Benjamín Díaz‐Ortiz, Cristóbal Baumann‐Biancani, Kamilo Hunger‐Abbott, Matías Herrera‐Matas, Andrés Vega‐Moraga, Vitor A. Lira, Maximiliano Espinosa‐Ramírez, Karol Ramírez‐Parada, Luigi Gabrielli‐Nervi, Hugo E. Verdejo, Felipe Contreras‐Briceño

**Affiliations:** ^1^ Laboratory of Exercise Physiology Department of Kinesiology School of Health Sciences Faculty of Medicine Pontificia Universidad Católica de Chile Santiago Chile; ^2^ Department of Informatics and Computing Faculty of Engineering Universidad Tecnológica Metropolitana Santiago Chile; ^3^ Department of Health and Human Physiology Fraternal Order of Eagles Diabetes Research Center College of Liberal Arts and Sciences University of Iowa Iowa City Iowa USA; ^4^ Exercise and Rehabilitation Sciences Institute, School of Physical Therapy Faculty of Rehabilitation Sciences, Pontificia Universidad Católica de Chile Santiago Chile; ^5^ Advanced Center for Chronic Diseases Hospital Clínico Pontificia Universidad Católica de Chile Santiago Chile; ^6^ Escuela de Kinesiología, Facultad de Salud Universidad Santo Tomás Santiago Chile

**Keywords:** biomedicine, exercise training, fatigue, near‐infrared spectroscopy, oxygen uptake

## Abstract

Biological sex influences exercise performance, largely owing to anatomical and physiological differences in brain areas involved in cognitive motor control and in respiratory and locomotor muscles related to workload. We used near‐infrared spectroscopy data to examine sex differences in haemodynamic responses and oxygenation patterns in the prefrontal cortex (PFC), m. intercostales and m. vastus lateralis during incremental exercise in 74 endurance‐trained individuals. Changes (Δ) in oxyhaemoglobin (O_2_‐Hb), deoxyhaemoglobin (H‐Hb) and tissue saturation index (TSI) were analysed using a two‐way ANOVA with the factors ‘sex’ and ‘intensity’. Effect sizes (ES) were also reported by partial eta squared (η_p_^2^). Interactions were observed for ΔO_2_‐Hb at the PFC [*p* *<* 0.001, η_p_^2^ *=* 0.42 (large ES)] and m. intercostales [*p* *<* 0.001, η_p_^2^ *=* 0.38 (large ES)], but not at m. vastus lateralis (*p* *=* 0.160). For ΔH‐Hb, interactions were observed at m. vastus lateralis [*p* *<* 0.001, η_p_^2^ *=* 0.35 (large ES)] and the PFC [*p* *=* 0.048, η_p_^2^ *=* 0.18 (large ES)]. The ΔTSI also showed an interaction at m. vastus lateralis [*p* *<* 0.001, η_p_^2^ *=* 0.44 (large ES)] and a trend in the m. intercostales (*p* *=* 0.057). Male subjects demonstrated greater oxygen delivery to the brain and increased peripheral deoxygenation, whereas females exhibited greater oxygen extraction in respiratory muscles, despite smaller body surface area. Higher tissue oxygen extraction reflects the capacity to meet local metabolic demands during exercise, enabling the identification of distinct oxygenation patterns between sexes. These findings suggest that sex‐specific mechanisms contribute to different patterns of physiological response to exercise. We support the hypothesis that peripheral factors might be more limiting in males, whereas in females central limitations (such as potential reduced oxygen delivery to the PFC owing to possible cerebral vasoconstriction triggered by metabolic reflexes) might play a more prominent role.

## Abstract (highlights)

- **What is the central question of this study?**
  Understanding the physiological differences between sexes is essential for addressing exercise limitations and developing targeted training strategies. Although research has highlighted variations in ventilatory function and muscle oxygenation, comprehensive evidence on how these differences affect physiological systems concurrently in endurance‐trained athletes during exercise has been little explored.
- **What is the main finding and its importance?**
  The integrative analysis of tissue oxygen levels using near‐infrared spectroscopy enabled us to identify the key biological systems that contribute to exercise intolerance among endurance‐trained athletes. Our findings revealed a sex‐specific pattern: female athletes predominantly exhibited limitations associated with central (brain‐related) mechanisms, whereas male athletes were more affected by peripheral (muscle‐related) mechanisms.

## Graphical abstract

**Abstract figure legend** During cardiopulmonary exercise testing, the decline in oxygenation in the prefrontal cortex of women is striking. This crucial brain area is directly involved in planning motor tasks. The decline is particularly pronounced at higher exercise intensities, especially after reaching the respiratory compensation point or anaerobic threshold, significantly influencing cognitive aspects of sports performance. In contrast, men are more affected by peripheral limiting factors due to the intense demands placed on their respiratory and locomotor muscle systems.

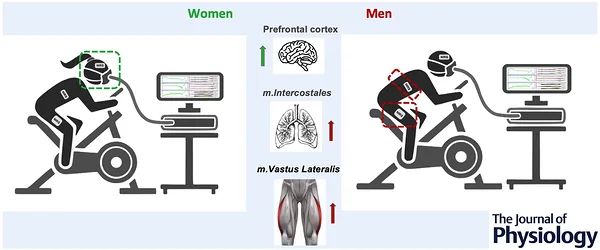

## 1 INTRODUCTION

Biological sex is a fundamental determinant of athletic performance, influencing physiological responses to exercise (Hunter et al., 2023). Despite the narrowing gap in sports participation, women have historically been underrepresented in athletic performance research. Consequently, current training methodologies are primarily derived from studies conducted on male participants (Costello et al., 2014), often overlooking anatomical and physiological differences specific to women that can influence the mechanisms limiting exercise performance.

In addition to biological sex, maximal oxygen uptake (${\dot{V}}_{O_{2}\max}$) is another key determining factor in athletic performance (Bassett, 2000). The ${\dot{V}}_{O_{2}\max}$ quantifies the maximal capacity of the body to deliver and utilize oxygen during strenuous exercise and can be expressed in both absolute values and relative to body mass (Jensen et al., 2001). Among elite endurance athletes, women exhibit lower relative ${\dot{V}}_{O_{2}\max}$ compared with their male counterparts (e.g., 67.1 ± 4.2 vs. 74.1 ± 2.6 mL kg^−1^ min^−1^; Pate & O'Neill, 2007), a difference attributed primarily to variations in body composition and oxygen‐carrying capacity. Sex differences in ${\dot{V}}_{O_{2}\max}$ also extend to the respiratory system, influencing ventilatory function during exercise. Women exhibit a higher oxygen uptake by the respiratory muscles compared with men (e.g., 13.8% ± 1.5% vs. 9.4% ± 1.1%, respectively; Dominelli et al., 2015), a difference attributed to anatomical and physiological factors. These include smaller lungs and a lower chest wall compliance, leading to lower lung volumes when in comparison to men of similar height and weight (Bellemare et al., 2003; LoMauro & Aliverti, 2018), in addition to narrower airways when adjusted for lung size (Sheel et al., 2016). These factors result in increased airway resistance and work of breathing for a given lung ventilation (${\dot{V}}_{E}$) (Santisteban et al., 2022; Sheel et al., 2016). This is reflected in a higher cost of breathing (Kipp et al., 2024) and greater deoxygenation of m. intercostales during intense exercise, whereas men exhibit greater deoxygenation of locomotor muscles (e.g., m. vastus lateralis), suggesting a higher peripheral workload during exercise (Espinosa‐Ramírez et al., 2021). To compensate, women rely more on accessory respiratory muscles during exercise (Mitchell et al., 2018).

This increased use of the respiratory muscles to meet ventilatory demands leads to greater oxygen uptake by these muscles during moderate to high levels of physical exertion (e.g., ${\dot{V}}_{E}$ > 65 L min^−1^). As a result, a greater proportion of cardiac output is redistributed to respiration in women compared with men (Guenette et al., 2007; Sheel et al., 2016). This phenomenon, known as the respiratory metaboreflex, reduces blood flow to the locomotor muscles, thereby limiting exercise capacity. Notably, men experience greater deoxygenation in locomotor muscles with increasing workload adjusted to weight, whereas women exhibit a more pronounced respiratory muscle deoxygenation per unit of ${\dot{V}}_{E}$, highlighting sex‐based differences in the trade‐off between respiratory and locomotor muscle oxygenation during exercise (Espinosa‐Ramírez et al., 2021).

The respiratory metaboreflex intensifies at exercise intensities close to the respiratory compensation point or the second ventilatory threshold (VT2, ∼85%–90% ${\dot{V}}_{O_{2}\max}$ ; Crisafulli et al., 2008). In these conditions, the elevated [H⁺] resulting from increased anaerobic metabolism leads to hyperventilation and, consequently, cerebral vasoconstriction secondary to hypocapnia. This can reduce blood flow to brain regions involved in cognitive motor control and exercise regulation, including the prefrontal cortex (PFC) (Querido & Sheel, 2007; Smith & Ainslie, 2017). The PFC, particularly the dorsolateral region, plays a critical role in executive functions, decision‐making, attention and cognitive control during complex motor tasks. Given the higher cost of breathing in women, this phenomenon is expected to be more pronounced, potentially contributing to the earlier onset of central limiting factors. In contrast, exercise limitation in men is more likely to originate from peripheral mechanisms.

To distinguish between central and peripheral patterns of oxygenation during exercise, the use of portable devices equipped with near‐infrared spectroscopy (NIRS) technology has recently been proposed as a monitoring tool (Carreño‐Román et al., 2024). This technology enables real time, non‐invasive assessment of tissue oxygenation dynamics by estimating changes in the concentrations of oxyhaemoglobin (O_2_‐Hb) and deoxyhaemoglobin (H‐Hb) (Barstow, 2019). It is important to note that continuous‐wave NIRS devices measure relative changes from baseline rather than absolute concentrations. The signal can also include contributions from the surrounding vasculature and skin blood flow, in addition to the target tissue. The combined signal from these two variables reflects total haemoglobin (T‐Hb). In addition, the tissue saturation index (TSI), calculated as the ratio of O_2_‐Hb to T‐Hb {([O_2_‐Hb]/[T‐Hb]) × 100}, indicates the balance between local oxygen delivery and utilization at the tissue level.

Given the anatomical and physiological differences between sexes, understanding their impact on exercise limitations is essential for developing targeted training strategies. Although previous research has described sex differences in ventilatory function and muscle oxygenation, there is limited integrative evidence on how these differences arise simultaneously in various physiological systems during exercise. To address this gap, in this cross‐sectional study we examined oxygenation dynamics in the PFC, m. intercostales and m. vastus lateralis during a cardiopulmonary exercise test (CPET) to exhaustion in male and female endurance athletes to determine whether these responses differ between sexes and to explore how changes in oxygenation are related to key physiological variables during exercise. We hypothesized that men would exhibit greater deoxygenation in the locomotor muscles owing to their higher peripheral metabolic demands, whereas women would show more pronounced deoxygenation in the respiratory muscles, reflecting their greater ventilatory cost of breathing. In turn, this ventilatory burden was expected to affect cerebral oxygenation, potentially compromising oxygen delivery in brain regions involved in cognitive motor control, such as the PFC. These oxygenation patterns were expected to be associated with distinct physiological responses between sexes, providing insight into the central versus peripheral oxygenation patterns influencing exercise performance.

## 2 MATERIALS AND METHODS

### 2.1 Participants

A total of 74 endurance‐trained individuals (44 men and 30 women) were recruited from July 2023 to November 2024 from university sports teams and recreational athletic clubs. Participants were screened for eligibility based on the following criteria: aged 19–45 years, with a body mass index of <25 kg m^−2^, and engagement in regular endurance training (at least three sessions per week, ≥30 min per session) for ≥6 months prior to enrolment. All individuals met the eligibility criteria and were enrolled in the study. Participants had no history of respiratory, cardiovascular, metabolic, musculoskeletal or neoplastic diseases. Additionally, none reported experiencing acute infections or inflammatory processes within the 2 weeks prior to testing. None of the participants followed a special diet or used any legal or illegal drugs. There were no exclusions or withdrawals; all enrolled participants (100%) completed the full protocol and all assessments involved in the study. All 74 participants were included in the final analysis, with no missing data. Each participant met at least three of the five established criteria for maximum effort during the CPET, confirming that the exercise tests reflected maximal exertion. Women were tested during the luteal phase of their menstrual cycle (days 15–28), based on self‐reported cycle tracking, and all participants reported having regular menstrual cycles ranging from 25 to 35 days. Data analysis was conducted by analysts who were blinded to the sex of the participant. The participant flow diagram is presented in the Supplementary material (Figure S1).

### 2.2 Sample size

The sample size was calculated using GPower™ (v.3.1.9.4) for a two‐way repeated‐measures ANOVA. We based the calculation on an expected effect size (ES) of *f* = 0.25, derived from previous studies examining sex differences in muscle oxygenation (Espinosa‐Ramírez et al., 2021). With a significance level (α) of 0.05, a power of 0.90, two groups, seven measurements, a correlation among repeated measures of 0.5 and a non‐sphericity correction (ε) of one, the required minimum sample size was estimated at 68 participants. To account for potential dropouts and data‐quality issues, we recruited 74 participants.

### 2.3 Ethical approval

The experimental protocol was approved by the Ethics Committee of Pontificia Universidad Católica de Chile (projects numbers 210525001 and 220608010), and the study was conducted in accordance with the principles outlined in the *Declaration of Helsinki*. This cross‐sectional study is reported following the Strengthening the Reporting of Observational Studies in Epidemiology (STROBE) guidelines (von Elm et al., 2008). All participants were informed beforehand about the procedures and gave written informed consent prior to their participation in the study.

### 2.4 Protocol

All assessments were conducted at the Laboratory of Exercise Physiology, Pontificia Universidad Católica de Chile, between 09.00 and 14.00 h in controlled laboratory conditions (temperature, 22°C ± 2°C and relative humidity, 40% ± 5%). Participants were instructed to abstain from physical activity, alcohol, caffeine and stimulants for 24 h prior to testing and to avoid food for ≥3 h before the evaluations. Upon arrival, anthropometric measurements and lung function assessments were performed, followed by the CPET.

### 2.5 Anthropometric and lung function assessment

Anthropometric characteristics, including weight, height and body mass index, were measured, and body surface area was calculated using the Mosteller formula (Mosteller, 1987). Lung function was assessed via spirometry (Microlab ML3500, CareFusion, San Diego, CA, USA), following the American Thoracic Society (ATS) and European Respiratory Society (ERS) guidelines (ATS/ERS, 2002), with Knudson reference values (Knudson et al., 1983). Additionally, maximal inspiratory pressure was measured using a pneumometer (Micro MRC, CareFusion, Traunstein, Germany) in accordance with ATS and ERS guidelines (Anon, 2002), using Black and Hyatt reference values (Black & Hyatt, 1969).

### 2.6 Cardiopulmonary exercise testing

The CPET was performed using an ergospirometer (Cosmed QUARK, Rome, Italy) and a ramp incremental protocol on a cycle ergometer (ViaSprint 150P, Ergoline GmbH, Traunstein, Germany). Exhaled gases, oxygen uptake (${\dot{V}}_{O_{2}}$), carbon dioxide production (${\dot{V}}_{CO_{2}}$) and ventilatory variables, including ${\dot{V}}_{E}$, tidal volume (*V*_T_) and respiratory rate (RR), were assessed using the breath‐by‐breath method. The protocol began with a 2 min unloaded rest period, followed by a 6 min warm‐up at a workload adjusted to body weight (0.6 W kg^−1^ for women and 0.8 W kg^−1^ for men). The exercise phase started at 80 W for women and 100 W for men, with subsequent increases of 20 and 25 W min^−1^, respectively, until exhaustion. Participants were required to maintain a cadence of 80–100 r.p.m., with failure to do so resulting in test termination. The protocol ended with a 3 min unloaded cool‐down phase. Maximum effort was confirmed when participants met at least three of the following five criteria: (1) respiratory exchange ratio (RER) ≥ 1.10; (2) heart rate ≥85% of age‐predicted maximum (220 − age); (3) plateau in ${\dot{V}}_{O_{2}}$ (<150 mL min^−1^ increase despite increasing workload); (4) rating of perceived exertion (RPE) ≥ 17 on the Borg scale (6–20); and (5) inability to maintain cadence of >60 r.p.m. despite strong verbal encouragement. All 74 participants met these criteria and completed the test to volitional exhaustion. Heart rate was monitored continuously (Quark T12x, Cosmed, Rome, Italy), and dyspnoea, lower‐limb fatigue and RPE were assessed using the modified Borg scale. Peak oxygen consumption (${\dot{V}}_{O_{2}\mathrm{peak}}$) was calculated as the highest value obtained during the last 30 s of CPET, despite increasing the exercise intensity (<150 mL min^−1^ of exercise) (Day et al., 2003). To complement the analysis, a triphasic model of exercise intensity was used, where ventilatory thresholds (AT (aerobic threshold) or VT1 (first ventilatory threshold) and respiratory compensation point or VT2) were determined by two expert exercise physiologists using the classic visual method (Guazzi et al., 2012), with no discrepancies between them.

### 2.7 Measurement of haemodynamic variables

Haemodynamic variables were monitored continuously in the PFC, accessory respiratory muscles (m. intercostales) and locomotor muscles (m. vastus lateralis) using NIRS devices (PortaLite and PortaMon; Artinis Medical Systems, Elst, The Netherlands). The changes (Δ), defined as the exercise intensity value minus the resting phase (baseline) value, in oxyhaemoglobin concentration ([O_2_‐Hb]) and deoxyhaemoglobin concentration ([H‐Hb]) were estimated. Additionally, total haemoglobin ([T‐Hb] = [O_2_‐Hb] + [H‐Hb]) and the TSI, calculated as ([O_2_‐Hb]/[T‐Hb] × 100), were calculated. Device placement followed a protocol published previously (Carreño‐Román et al., 2024). Briefly, for the PFC, a PortaLite probe was positioned on the left dorsolateral prefrontal cortex (Brodmann area 9/46), ∼10 mm above the superciliary arch. This region is involved in executive functions, working memory and cognitive control during exercise, rather than direct motor unit recruitment. For the m. intercostales, a second PortaLite probe was placed at the right anterior axillary line over the seventh intercostal space (Vogiatzis et al., 2009). Finally, for the m. vastus lateralis, a PortaMon probe was positioned 5 cm lateral to the midpoint of the imaginary line connecting the upper edge of the patella and the greater trochanter of the right femur. The position of a NIRS device at m. intercostales and m. vastus lateralis has been reported previously (Contreras‐Briceño et al., 2022; Espinosa‐Ramírez et al., 2021), with good reliability (Contreras‐Briceño et al., 2019; Sendra‐Pérez et al., 2023).

### 2.8 Data analysis

The NIRS data were recorded at 10 Hz using software provided by the manufacturer (Oxysoft, Artinis Medical Systems, Elst, The Netherlands). After data acquisition, a 10th order low‐pass zero‐phase Butterworth filter (cut‐off frequency, 0.1 Hz) was applied to reduce artefacts and smooth signal fluctuations, following a previous study that assessed the same tissues using identical devices (Woorons et al., 2019). The changes (Δ) in [O_2_‐Hb] and [H‐Hb], expressed in micromolar units, were calculated by subtracting the mean values at each exercise intensity from baseline. Baseline values for each variable were defined as the mean of the 10 s preceding and following the midpoint between the start of data acquisition and the onset of the warm‐up phase. To determine NIRS values at different exercise intensities (expressed as percentages of ${\dot{V}}_{O_{2}\mathrm{peak}}$ ranging from 40% to 100%) the mean of the 20 s prior to each intensity percentage, based on the CPET variables, was used. The differential pathlength factor was set to six for the PFC and four for the respiratory and locomotor muscles, in accordance with previous studies in athletes (Aebi et al., 2019; Carreño‐Román et al., 2024; Cocking et al., 2021). Changes from baseline were reported as either positive or negative values, indicating increases or decreases, respectively.

### 2.9 Statistical analysis

The normal distribution was tested using the Shapiro–Wilk test. Sex differences in anthropometric and lung function variables were analysed using Student's unpaired *t*‐test. Effect sizes for comparison between groups were calculated using Cohen's *d*, with values interpreted as small (0.20 < |*d| *< 0.50), medium (0.50 < |*d| *< 0.80), large (0.80 < |*d| *< 1.20) or very large (|*d|* > 1.20) according to established benchmarks (Cohen, 2013; Sawilowsky, 2009). To test whether the haemodynamic variables from NIRS data were affected by the factors ‘sex’ and ‘intensity’, we used two‐way ANOVA with ‘sex’ (levels: women and men) and ‘intensity’ (levels: 40%, 50%, 60%, 70%, 80%, 90% and 100% of ${\dot{V}}_{O_{2}\mathrm{peak}}$) as independent factors and NIRS data as the dependent factor with Tukey's *post hoc* test. Effect sizes were calculated as partial eta squared (η_p_^2^) for all ANOVAs, with values interpreted as small (0.01 < η_p_^2^ *<* 0.06), medium (0.06 < η_p_^2^ *<* 0.14) or large (η_p_^2^ > 0.14) according to Cohen's guidelines (Cohen, 2013). The Pearson correlation test was used to analyse the associations between NIRS data and physiological variables assessed during CPET. Although multiple comparisons across tissues and variables increase the risk of type I error, we did not apply additional corrections beyond Tukey's *post hoc* test, because our primary hypotheses were directional and based on strong a priori physiological rationale. However, we emphasize effect sizes alongside *p*‐values to provide a more complete picture of the observed differences. Statistical significance was set at *p* < 0.05. All statistical analyses were performed using GraphPad Prism (v.10.; San Diego, CA, USA).

## 3 RESULTS

All recruited participants finished the study; i.e., there was no attrition. Female athletes reported regular menstrual cycles and were evaluated during the luteal phase. According to Shapiro–Wilk and Levene's tests, the normality and homoscedasticity assumptions were not violated.

### 3.1 Characteristics of participants

Anthropometric and lung function data are presented in Table 1. Significant sex differences were observed in all variables except age.

**TABLE 1:** Characteristics of participants (*n* = 74).

Characteristic	Male (*n* = 44)	Female (*n* = 30)	*P*‐value	ES
Age, years	25.1 ± 7.3	22.6 ± 4.4	n.s.	0.41^†^
Height, cm	175.7 ± 4.6^*^	165.5 ± 6.2	<0.001	1.90^‡^
Weight, kg	72.8 ± 7.2^*^	58.2 ± 6.1	<0.001	2.19^†^
BMI, kg m^−2^	23.6 ± 2.2^*^	21.2 ± 2.0	<0.001	1.14^†^
BSA, m^2^	1.88 ± 0.10^*^	1.63 ± 0.10	<0.001	2.50^†^
MIP, cmH_2_O	137.6 ± 23.6^*^	118.6 ± 32.6	<0.010	0.68^†^
MIP, % predicted	106.7 ± 18.9^*^	128.4 ± 35.0	<0.001	0.79^‡^
FEV_1_, L	4.4 ± 0.6^*^	3.4 ± 0.4	<0.001	1.96^†^
FEV_1_, % predicted	104.1 ± 13.2^*^	84.8 ± 11.3	<0.001	1.56^†^
TLC, L	5.1 ± 0.3^*^	4.6 ± 0.3	<0.001	1.67^†^
TLC, % predicted	106.3 ± 11.9^*^	100.6 ± 9.5	<0.050	0.53^†^
FEV_1_/TLC	81.3 ± 7.0^*^	85.7 ± 9.5	<0.050	0.53^‡^

*Note*: Data are expressed as the mean ± SD. Abbreviations: BMI, body mass index; BSA, body surface area; ES, effect size calculated as Cohen's *d*, absolute value; FEV_1_, forced expiratory volume in first second; MIP, maximal inspiratory pressure; TLC, total lung capacity.

* *p* < 0.05 (Student's t‐test).

^†^ Males > females.

^‡^ Females > males.

To ensure the integrity of the research, all analyses were performed blindly, meaning that the data scientists analysing the NIRS data were unaware of the sex of participants.

### 3.2 Changes in physiological variables measured by ergospirometer during CPET

The comparison of physiological variables between sexes during CPET is presented in Table 2. Significant interaction differences were observed for all variables, except for respiratory rate.

**TABLE 2:** Comparison between physiological variables obtained during cardiopulmonary exercise testing (*n* = 74).

Variables	Males (*n* = 44) / Women (*n* = 30)								
	$\%{\dot{V}}_{o_{2}\mathrm{peak}}$							AT	RCP
	40%	50%	60%	70%	80%	90%	100%		
$\Delta {\dot{V}}_{o_{2}}$, mL kg^−1^ min^−1^ Interaction: *p <* 0.001 sex: *p <* 0.001 intensity: *p <* 0.001 η_p_^2^ *=* 0.42	20.0 ± 2.3/18.1 ± 3.0	25.1 ± 2.8/22.6 ± 3.9^*^	30.0 ± 3.6/27.2 ± 4.5^*^	35.1 ± 4.2/31.7 ± 5.4^*^	40.0 ± 4.8/36.1 ± 6.0^*^	45.1 ± 5.2/40.9 ± 6.8^*^	50.1 ± 5.9/45.2/7.6^*^	26.6 ± 4.5/26.6/6.6	43.6 ± 5.5/39.9 ± 7.5^*^
${\dot{V}}_{E}/{\dot{V}}_{CO_{2}}$, L min^−1^ Interaction: *p *< 0.001 sex: *p <* 0.001 intensity: *p <* 0.001 η_p_^2^ *=* 0.38	1.43 ± 0.16/1.04 ± 0.16^*^	1.82 ± 0.21/1.30 ± 0.20^*^	2.17 ± 0.24/1.57 ± 0.24^*^	2.54 ± 0.29/1.83 ± 0.28^*^	2.90 ± 0.34/2.08 ± 0.32^*^	3.27 ± 0.38/2.36 ± 0.36^*^	3.63 ± 0.42/2.61 ± 0.40^*^	1.92 ± 0.32/1.53 ± 0.32^*^	3.16 ± 0.41/2.30 ± 0.40^*^
${\dot{V}}_{CO_{2}}$, L min^−1^ Interaction: *p <* 0.001 sex: *p <* 0.001 intensity: *p <* 0.001 η_p_^2^ *=* 0.45	1.22 ± 0.17/0.81 ± 0.16^*^	1.60 ± 0.22/1.08 ± 0.16^*^	2.01 ± 0.24/1.35 ± 0.22^*^	2.49 ± 0.27/1.70 ± 0.28^*^	2.95 ± 0.30/2.06 ± 0.32^*^	3.46 ± 0.31/2.45 ± 0.34^*^	4.07 ± 0.35/2.86 ± 0.41^*^	1.69 ± 0.33/1.30 ± 0.32^*^	3.30 ± 0.37/2.36 ± 0.40^*^
RQ Interaction: *p =* 0.002 sex: *p <* 0.001 intensity: *p =* 0.008 η_p_^2^ *= 0.18*	0.92 ± 0.08/0.77 ± 0.07^*^	0.98 ± 0.09/0.82 ± 0.04^*^	0.93 ± 0.08/0.86 ± 0.04^*^	0.98 ± 0.09/0.93 ± 0.07^*^	1.02 ± 0.08/0.99 ± 0.05	1.06 ± 0.08/1.04 ± 0.06	1.12 ± 0.08/1.10 ± 0.06	0.87 ± 0.08/0.85 ± 0.07	1.05 ± 0.07/1.02 ± 0.06
${\dot{V}}_{E}$, L Interaction: *p <* 0.001 sex: *p <* 0.001 intensity: *p <* 0.001 η_p_^2^ *=* 0.52	35 ± 7/25 ± 4^*^	43 ± 7/32 ± 4^*^	53 ± 7/38 ± 6^*^	65 ± 8/47 ± 8^*^	78 ± 10/58 ± 11^*^	98 ± 13/72 ± 13^*^	139 ± 21/95 ± 20^*^	44 ± 8/37 ± 10^*^	91 ± 15/68 ± 15^*^
${\dot{V}}_{E}/\mathrm{BSA}$ Interaction: *p <* 0.001 sex: *p <* 0.001 intensity: *p <* 0.001 η_p_^2^ *= 0.35*	18.5 ± 3.4/15.7 ± 2.7	22.9 ± 3.3/19.8 ± 2.9^*^	28.1 ± 3.3/23.5 ± 4.2^*^	34.5 ± 4.3/18.9 ± 5.1^*^	41.8 ± 4.8/35.7 ± 7.0^*^	52.4 ± 6.6/44.3 ± 8.2^*^	73.7 ± 10.8/58.3 ± 12.4^*^	23.5 ± 4.2/22.7 ± 6.9	48.3 ± 7.4/41.6 ± 9.5^*^
*V*_T_, L Interaction: *p <* 0.001 sex: *p <* 0.001 intensity: *p <* 0.001 η_p_^2^ *= 0.28*	2.11 ± 0.83/1.35 ± 0.40^*^	2.28 ± 0.77/1.56 ± 0.42^*^	2.49 ± 0.48/1.74 ± 0.38^*^	2.78 ± 0.71/1.89 ± 0.29^*^	2.96 ± 0.56/1.97 ± 0.25^*^	3.06 ± 0.58/2.11 ± 0.28^*^	3.08 ± 0.59/2.09 ± 0.24^*^	2.34 ± 0.58/1.72 ± 0.42^*^	3.09 ± 0.55/2.080.28^*^
RR (breaths min^−1^) Interaction: *p =* 0.219 sex: *p =* 0.128 intensity: *p <* 0.001 η_p_^2^ *=* 0.08	17 ± 4/20 ± 4	20 ± 4/21 ± 4	22 ± 4/23 ± 4	25 ± 5/25 ± 4	27 ± 4/29 ± 5	32 ± 5/34 ± 6	46 ± 9/45 ± 8	19 ± 4/22 ± 5	30 ± 5/32 ± 7^*^
${\dot{V}}_{E}/{\dot{V}}_{o_{2}}$ Interaction: *p =* 0.004 sex: *p =* 0.950 intensity: *p <* 0.001 η_p_^2^ *= 0.16*	23.0 ± 3.5/23.4 ± 2.2	23.0 ± 2.4/23.6 ± 1.9	23.7 ± 2.1/23.8 ± 2.0	25.0 ± 2.4/24.8 ± 2.3	26.5 ± 2.5/26.8 ± 2.8	29.6 ± 3.2/29.6 ± 3.8	37.5 ± 5.3/35.3 ± 5.4^*^	22.2 ± 2.1/23.0 ± 2.6	28.1 ± 3.2/28.4 ± 3.6
${\dot{V}}_{E}/{\dot{V}}_{CO_{2}}$ Interaction: *p <* 0.001 sex: *p =* 0.083 intensity: *p <* 0.001 η_p_^2^ *=* 0.22	27.5 ± 4.4/30.2 ± 2.2^*^	26.3 ± 3.1/28.5 ± 2.1^*^	25.6 ± 3.0/27.2 ± 2.0^*^	25.5 ± 2.6/26.7 ± 2.3	26.1 ± 2.7/27.1 ± 2.9	27.9 ± 3.3/28.4 ± 3.4	33.3 ± 4.9/32.0 ± 5.0	25.9 ± 3.0/27.1 ± 2.4^*^	26.8 ± 3.1/27.8 ± 3.6
$P_{\mathrm{ET},O_{2}}$, mmHg Interaction: *p =* 0.006 sex: *p =* 0.476 intensity: *p <* 0.001 η_p_^2^ *=* 0.15	93.9 ± 5.4/95.2 ± 2.0	93.9 ± 4.0/95.3 ± 3.8	94.8 ± 3.8/95.1 ± 3.7	96.8 ± 3.7/97.0 ± 3.6	98.9 ± 3.4/99.7 ± 3.7	102.7 ± 4.0/102.9 ± 4.3	109.4 ± 3.9/107.8 ± 4.6	92 ± 4/94.2 ± 3.9	100 ± 5/101.5 ± 4.3
$P_{\mathrm{ET},CO_{2}}$, mmHg Interaction: *p <* 0.001 sex: *p =* 0.020 intensity: *p <* 0.001 η_p_^2^ *= 0.24*	40.6 ± 5.1/36.4 ± 2.8^*^	42.0 ± 4.3/38.7 ± 3.1^*^	43.2 ± 4.5/40.2 ± 3.1^*^	43.5 ± 4.1/41.2 ± 3.8^*^	42.8 ± 3.9/40.8 ± 4.3	40.6 ± 4.5/39.2 ± 4.5	34.7 ± 4.8/35.6 ± 5.0	43 ± 4/40.7 ± 3.4^*^	42 ± 4/40.3 ± 4.9
HR (beats min^−1^) Interaction: *p =* 0.005 sex: *p =* 0.391 intensity: *p <* 0.001 η_p_^2^ *= 0.14*	109 ± 13/109 ± 14	122 ± 12/121 ± 16	135 ± 13/132 ± 15	149 ± 12/146 ± 16	162 ± 11/158 ± 15	170 ± 10/165 ± 16	174 ± 12/167 ± 17^*^	125 ± 15/130 ± 16	168 ± 11/162 ± 17
%HR_max_, (220 − age) Interaction: *p =* 0.001 sex: *p =* 0.106 intensity: *p <* 0.001 η_p_^2^ *=* 0.19	56 ± 6/55 ± 7	62 ± 6/61 ± 8	69 ± 6/67 ± 7	76 ± 5/74 ± 7	83 ± 5/80 ± 7	87 ± 4/83 ± 8^*^	90 ± 7/85 ± 9^*^	64 ± 8/66 ± 8	86 ± 5/82 ± 9^*^
Workload (W) Interaction: *p <* 0.001 sex: *p <* 0.001 intensity: *p <* 0.001 η_p_^2^ *=* 0.58	78 ± 36/38 ± 20^*^	120 ± 44/74 ± 27^*^	157 ± 39/103 ± 29^*^	198 ± 45/134 ± 25^*^	229 ± 42/165 ± 27^*^	262 ± 40/192 ± 26^*^	300 ± 41/220 ± 35^*^	128 ± 47/99 ± 39^*^	253 ± 42/183 ± 33^*^
Workload (W kg^−1^) Interaction: *p =* 0.003 sex: *p =* 0.013 intensity: *p <* 0.001 η_p_^2^ *= 0.17*	1.07 ± 0.51/0.67 ± 0.28^*^	1.66 ± 0.61/1.30 ± 0.49^*^	2.18 ± 0.55/1.80 ± 0.55^*^	2.73 ± 0.63/2.33 ± 0.47^*^	3.16 ± 0.58/2.86 ± 0.52^*^	3.61 ± 0.58/3.33 ± 0.48^*^	4.14 ± 0.60/3.80 ± 0.67^*^	1.77 ± 0.65/1.75 ± 0.77	3.48 ± 0.58/3.17 ± 0.63^*^

*Note*: Data are expressed as mean ± SD. Abbreviations: η_p_^2^, effect size (partial eta squared) calculated for all two‐way ANOVA interactions (‘sex’ × ‘intensity’); AT, aerobic threshold; HR, heart rate; $P_{\mathrm{ET},CO_{2}}$, end‐tidal partial pressure of carbon dioxide; $P_{\mathrm{ET},O_{2}}$, end‐tidal partial pressure of oxygen; RCP, respiratory compensation point; RQ, respiratory quotient (${\dot{V}}_{CO_{2}}/{\dot{V}}_{O_{2}}$); RR, respiratory rate; ${\dot{V}}_{CO_{2}}$ , carbon dioxide production; ${\dot{V}}_{E}$, lung ventilation; ${\dot{V}}_{E}/\mathrm{BSA}$, lung ventilation per body surface area; ${\dot{V}}_{O_{2}}$ , oxygen uptake; *V*_T_, tidal volume; ${\dot{V}}_{E}/{\dot{V}}_{CO_{2}}$, ventilatory equivalent of carbon dioxide; ${\dot{V}}_{E}/{\dot{V}}_{O_{2}}$, ventilatory equivalent of oxygen.

* Statistical difference between‐sex effect (two‐way ANOVA test).

### 3.3 Prefrontal cortex haemodynamics and oxygenation during exercise

Figure 1 illustrates the changes (Δ) in haemodynamic variables in the PFC, a brain region involved in cognitive motor control and executive functions during exercise. For ΔO_2_‐Hb (Figure 1a), a significant interaction was observed (*p* *<* 0.001, η_p_^2^ *=* 0.42, large ES). Both sexes showed increased ΔO_2_‐Hb with increasing exercise intensity; however, male subjects exhibited a continuous increase up to exhaustion, whereas females displayed a slight decline at 100% of ${\dot{V}}_{O_{2}\mathrm{peak}}$ compared with 90%. The ΔO_2_‐Hb values for male subjects were significantly higher than those of females from 60% to 100% of ${\dot{V}}_{O_{2}\mathrm{peak}}$ (*p* *<* 0.050). For ΔH‐Hb (Figure 1b), no interaction or sex differences were found. Both sexes demonstrated a gradual increase across intensities, indicating increased oxygen extraction. Notably, male subjects showed a significant increase in ΔH‐Hb from baseline at high intensities (≥90% ${\dot{V}}_{O_{2}\mathrm{peak}}$, *p* *<* 0.050), whereas this was not observed in females. The ΔT‐Hb (Figure 1c) also showed a significant interaction (*p* *<* 0.001). In both sexes, ΔT‐Hb increased with intensity, with significant changes from baseline observed at ≥60% of ${\dot{V}}_{O_{2}\mathrm{peak}}$ (*p* *<* 0.05). Given that this parameter is associated with blood volume or blood flow, it suggests that as exercise intensity rises and more motor units are recruited, the PFC requires greater oxygen and nutrient supply for motor planning. Additionally, from 60% to 100% of ${\dot{V}}_{O_{2}\mathrm{peak}}$, male subjects exhibited significantly higher ΔT‐Hb values than females (*p* *<* 0.05). Finally, ΔTSI (Figure 1d) also showed a significant interaction (*p* *<* 0.001). A biphasic pattern was observed in both groups: in male subjects, ΔTSI rose up to 60% of ${\dot{V}}_{O_{2}\mathrm{peak}}$ before declining at higher intensities, whereas in females it increased more gradually up to 80%, followed by a plateau and slight decrease at 100%. This trend might reflect sex differences in the balance between oxygen delivery and extraction in the PFC during intense exercise.

**FIGURE 1:**
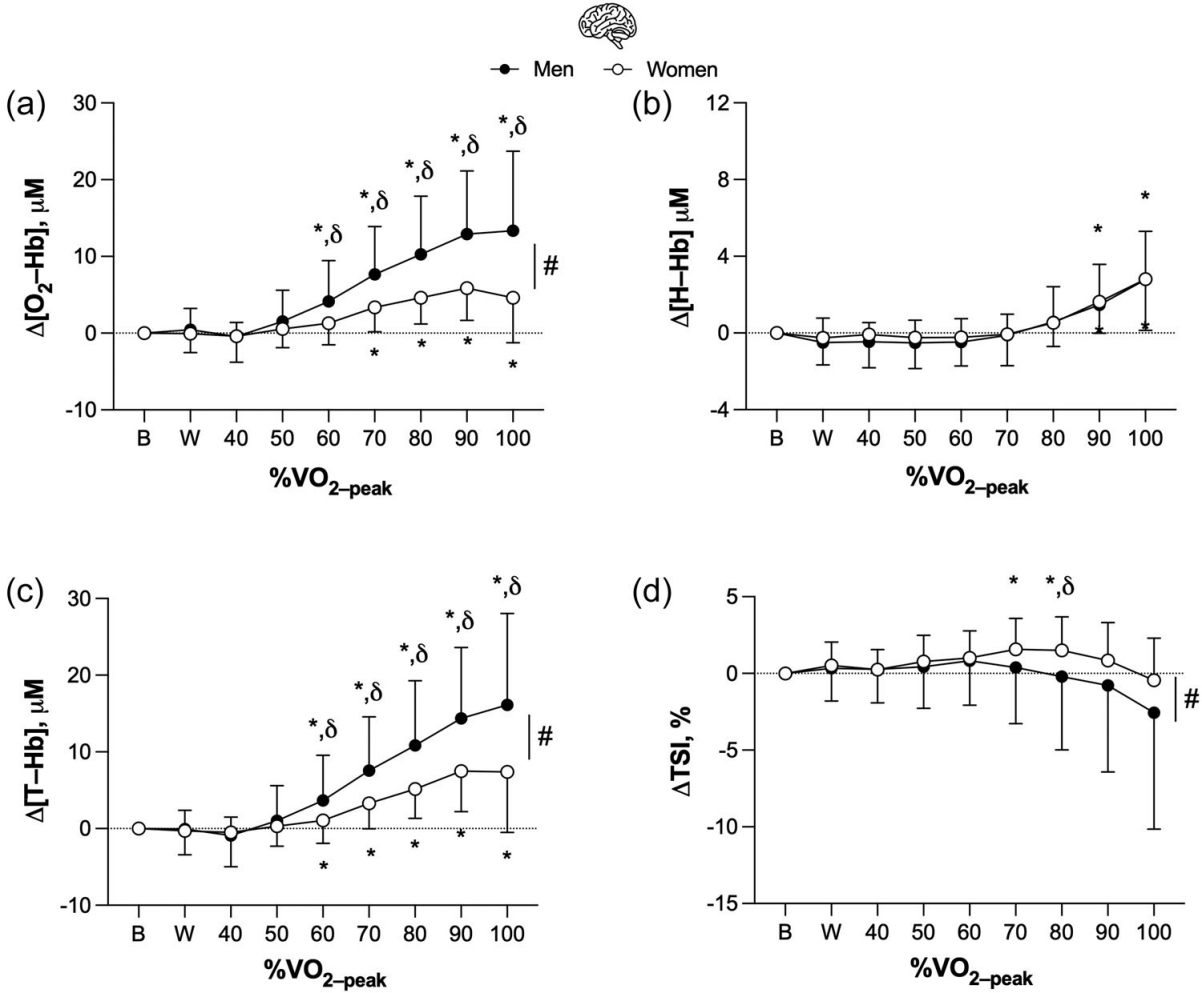
Sex comparison of changes (Δ) in near‐infrared spectroscopy data from the prefrontal cortex during cardiopulmonary exercise testing (*n* = 74): (a) oxyhaemoglobin (Δ[O_2_‐Hb]); (b) deoxyhaemoglobin (Δ[H‐Hb]); (c) total haemoglobin (Δ[T‐Hb]); and (d) tissue saturation index (ΔTSI). ^#^ *p* *<* 0.05, interaction of factors (‘sex’ × ‘intensity’). ^*^ *p* *<* 0.05, difference versus baseline phase in factor ‘intensity’. ^δ^ *p* *<* 0.05 difference in factor ‘sex’.

### 3.4 Respiratory muscle haemodynamics and oxygenation during exercise (m. intercostales)

Figure 2 illustrates the changes in haemodynamic variables measured in the accessory respiratory muscles, which are actively recruited during exercise and contribute to the cost of breathing as ventilatory demands increase. For ΔO_2_‐Hb (Figure 2a), a significant interaction was observed (*p* *<* 0.001). Both sexes exhibited a decline in ΔO_2_‐Hb as exercise intensity increased; however, this decrease was more pronounced in female subjects, who showed reductions from the onset of exercise. In contrast, males maintained relatively stable values until 90% of ${\dot{V}}_{O_{2}\mathrm{peak}}$, after which a marked decline was observed up to exhaustion (*p* *<* 0.05). Significant sex differences were present throughout the exercise test. For ΔH‐Hb (Figure 2b), no significant interaction was found. Both sexes demonstrated similar overall trends, with ΔH‐Hb increasing as exercise intensity rose. However, female subjects showed significantly higher ΔH‐Hb values from the beginning of exercise until 90% of ${\dot{V}}_{O_{2}\mathrm{peak}}$, suggesting a greater reliance on oxygen extraction in the respiratory muscles under rising ventilatory demands. For ΔT‐Hb (Figure 2c), a significant interaction was observed (*p* *<* 0.001). In male subjects, ΔT‐Hb increased up to 80% of ${\dot{V}}_{O_{2}\mathrm{peak}}$ before declining at higher intensities. In contrast, female subjects showed a slight increase up to 40% of ${\dot{V}}_{O_{2}\mathrm{peak}}$, followed by a gradual decrease, reaching significantly lower values at 100% of ${\dot{V}}_{O_{2}\mathrm{peak}}$ (*p* *<* 0.05). These sex differences are likely to reflect the interplay between oxygen delivery and extraction shown in Figure 2a, b. Lastly, for ΔTSI (Figure 2d), no significant interaction was found. Both sexes exhibited a significant decline from 40% of ${\dot{V}}_{O_{2}\mathrm{peak}}$ to exhaustion, with the reduction becoming more pronounced at higher intensities. This pattern is likely to reflect increasing ventilatory demands and systemic metabolic stress beyond VT2.

**FIGURE 2:**
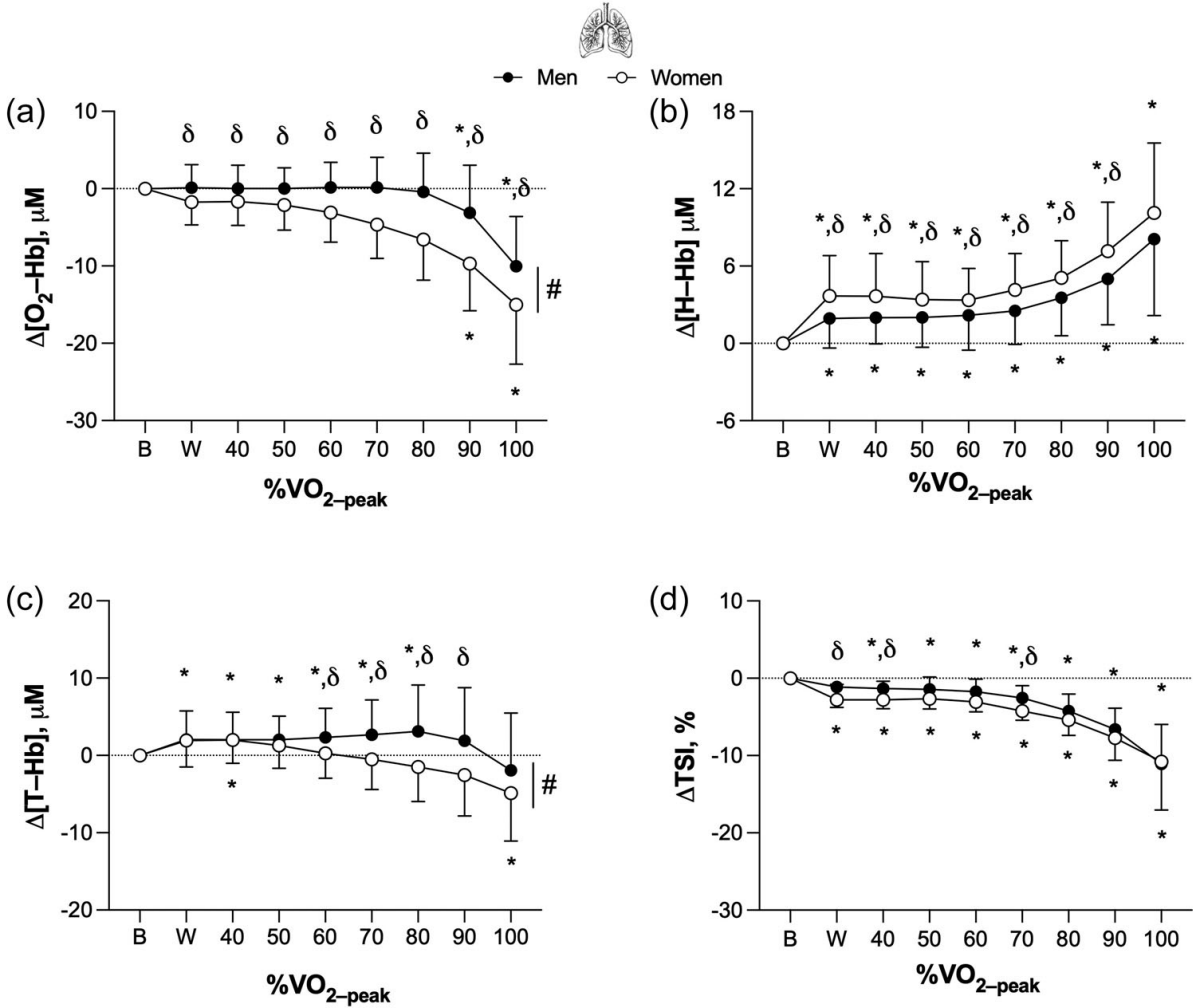
Sex comparison of changes (Δ) in near‐infrared spectroscopy data from the m. intercostales during cardiopulmonary exercise testing (*n* = 74): (a) oxyhaemoglobin (Δ[O_2_‐Hb]); (b) deoxyhaemoglobin (Δ[H‐Hb]); (c) total haemoglobin (Δ[T‐Hb]); and (d) tissue saturation index (ΔTSI). ^#^ *p* *<* 0.05, interaction of factors (‘sex’ × ‘intensity’). ^*^ *p* *<* 0.05, difference versus baseline phase in factor ‘intensity’. ^δ^ *p* *<* 0.05, difference in factor ‘sex’.

### 3.5 Locomotor muscle haemodynamics and oxygenation during exercise (m. vastus lateralis)

Figure 3 shows the changes in haemodynamic variables assessed in the primary locomotor muscles recruited during repetitive and cyclic contractions, such as the ones involved during the CPET protocol. These changes reflect the metabolic stress induced by exercise. For ΔO_2_‐Hb (Figure 3a), no significant interaction was observed. Furthermore, both sexes exhibited similar trends, with a significant decline in ΔO_2_‐Hb from 40% up to 100% of ${\dot{V}}_{O_{2}\mathrm{peak}}$. In contrast, a significant interaction was found for ΔH‐Hb (Figure 3b; *p* *<* 0.001). Throughout CPET, male subjects consistently showed higher values of ΔH‐Hb than females. Additionally, male subjects exhibited a significant and gradual increase in ΔH‐Hb beginning at 40% of ${\dot{V}}_{O_{2}\mathrm{peak}}$, whereas the values for female subjects remained relatively stable. This suggests that men extract more oxygen in the locomotor muscles, possibly owing to the higher initial workloads at which they began the CPET protocol. For ΔT‐Hb (Figure 3c), a significant interaction was also observed (*p* *<* 0.001), with distinct sex‐specific trends. In male subjects, ΔT‐Hb increased significantly from 50% of ${\dot{V}}_{O_{2}\mathrm{peak}}$ up to exhaustion, with a slight decline from 90% to 100%. In contrast, female subjects showed a continuous decrease in ΔT‐Hb from the onset of exercise to exhaustion. These responses differed significantly at each intensity, except at 50% of ${\dot{V}}_{O_{2}\mathrm{peak}}$ (*p* *<* 0.05). These patterns suggest that men are better able to maintain or increase blood volume delivery to the locomotor muscles during sustained contractions, whereas women exhibit a relative decline despite increasing metabolic demands. Finally, ΔTSI (Figure 3d) also demonstrated a significant interaction. Although both sexes demonstrated decreasing trends, male subjects exhibited significantly lower ΔTSI values than females from the beginning of exercise (*p* *<* 0.050). This difference became more pronounced at higher intensities, reaching values ∼12% lower. Considering that the decline in ΔTSI in male subjects was significant from the onset of exercise to exhaustion (*p* *<* 0.050), it can be inferred that men experience greater deoxygenation of the locomotor muscles, particularly at higher intensities. As noted previously, this might be explained, in part, by the higher absolute workload experienced by male subjects during the CPET protocol.

**FIGURE 3:**
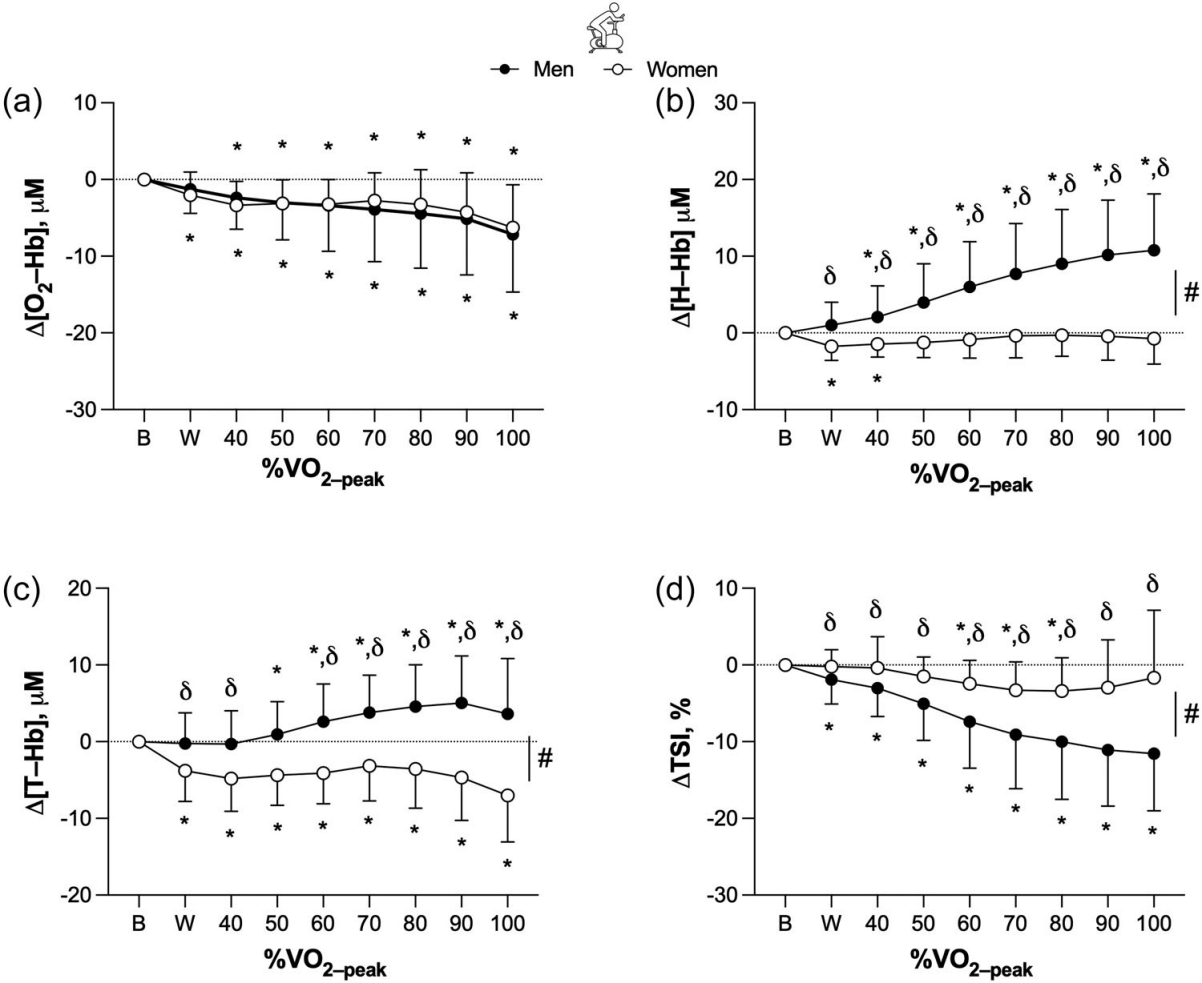
Sex comparison of changes (Δ) in near‐infrared spectroscopy data from the m. vastus lateralis during cardiopulmonary exercise testing (*n* = 74): (a) oxyhaemoglobin (Δ[O_2_‐Hb]); (b) deoxyhaemoglobin (Δ[H‐Hb]); (c) total haemoglobin (Δ[T‐Hb]); and (d) tissue saturation index (ΔTSI). ^#^ *p* *<* 0.05, interaction of factors (‘sex’ × ‘intensity’). ^*^ *p* *<* 0.05, difference versus baseline phase in factor ‘intensity’. ^δ^ *p* *<* 0.05, difference in factor ‘sex’.

### 3.6 Changes in ventilatory variables during CPET

Given the anatomical differences in thoracic structure between sexes, changes in lung ventilation (${\dot{V}}_{E}$) were adjusted for body surface area (ratio V˙E/V˙EBSABSA), a variable used previously because it is considered a better metabolic mass indicator (Araneda et al., 2018; Contreras‐Briceño et al., 2020). Additionally, changes in end‐tidal partial pressure of carbon dioxide ($P_{\mathrm{ET},CO_{2}}$), measured via ergospirometry during CPET, were analysed owing to consistent evidence that changes in this parameter can influence cerebral blood flow (Querido & Sheel, 2007; Smith & Ainslie, 2017).

Figure 4 presents changes occurring during the exercise phase of the CPET protocol (from 40% to 100% of ${\dot{V}}_{O_{2}\mathrm{peak}}$), showing a significant interaction. Male subjects exhibited higher V˙E/V˙EBSABSA values than females, accompanied by a more pronounced decrease in $P_{\mathrm{ET},CO_{2}}$. This pattern of hyperventilation and resulting hypocapnia might contribute to the observed reductionin Δ[O_2_‐Hb] in PFC.

**FIGURE 4:**
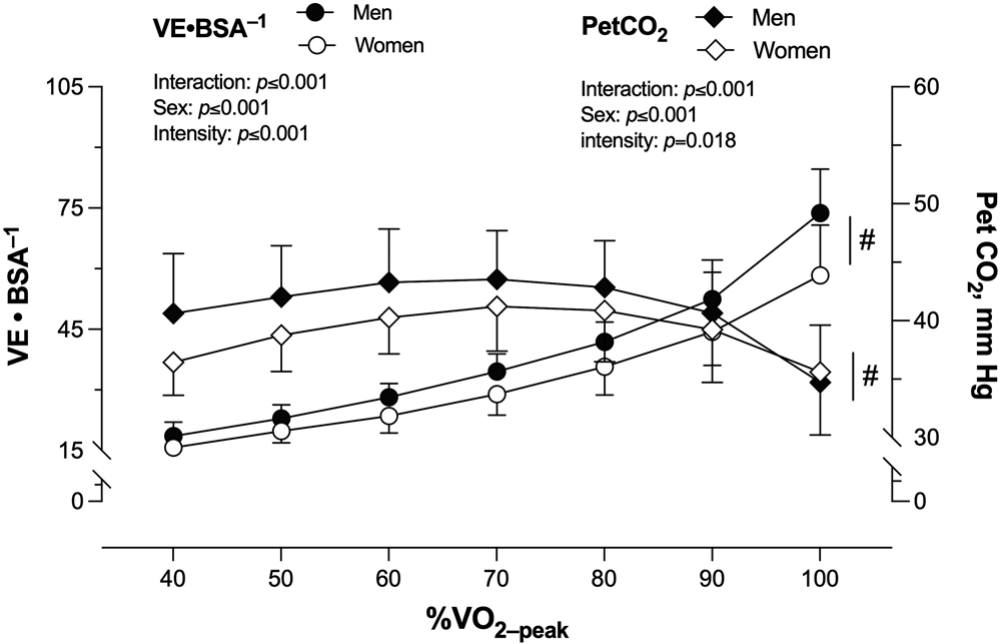
Sex comparison of changes (Δ) in ventilatory variables assessed during cardiopulmonary exercise testing (*n* = 74). Abbreviations: $P_{\mathrm{ET},CO_{2}}$, end‐tidal partial pressure of carbon dioxide; V˙E/V˙EBSABSA, the ratio between lung ventilation (${\dot{V}}_{E}$) and body surface area (BSA). ^#^ *p <* 0.05, interaction of factors (‘sex’ × ‘intensity’).

### 3.7 Associations

To examine whether changes in tissue oxygenation (ΔTSI) are associated with systemic metabolic demand ($\Delta {\dot{V}}_{O_{2}}$) and ventilatory demand adjusted for body surface area (ΔV˙E/V˙EBSABSA) during incremental exercise, we performed correlation analyses across all participants. The purpose was to determine whether tissue desaturation is related to increases in oxygen consumption (reflecting local metabolic stress) and whether ventilation, which might trigger metaboreflex‐mediated blood flow redistribution, affects tissue oxygenation in a tissue‐specific manner.

Figure 5 illustrates the correlations between ΔTSI during the exercise phase of CPET and changes in aerobic capacity ($\Delta {\dot{V}}_{O_{2}}$, in litres per minute) and the ΔV˙E/V˙EBSABSA ratio across all participants. The ΔTSI was inversely associated with $\Delta {\dot{V}}_{O_{2}}$ in all three assessed tissues: prefrontal cortex (Figure 5a), m. intercostales (Figure 5b) and m. vastus lateralis (Figure 5c). These negative correlations indicate that greater metabolic demand is associated with increased tissue oxygen extraction. In addition, ΔTSI showed a direct association with ΔV˙E/V˙EBSABSA in the prefrontal cortex (Figure 5d) and m. vastus lateralis (Figure 5f). This positive association suggests that tissues with higher TSI values maintain better oxygenation despite increasing ventilatory demands. Interestingly, ΔTSI was not significantly associated with ΔV˙E/V˙EBSABSA in m. intercostales, although a negative trend was observed (*r* = −0.22, *p =* 0.059).

**FIGURE 5:**
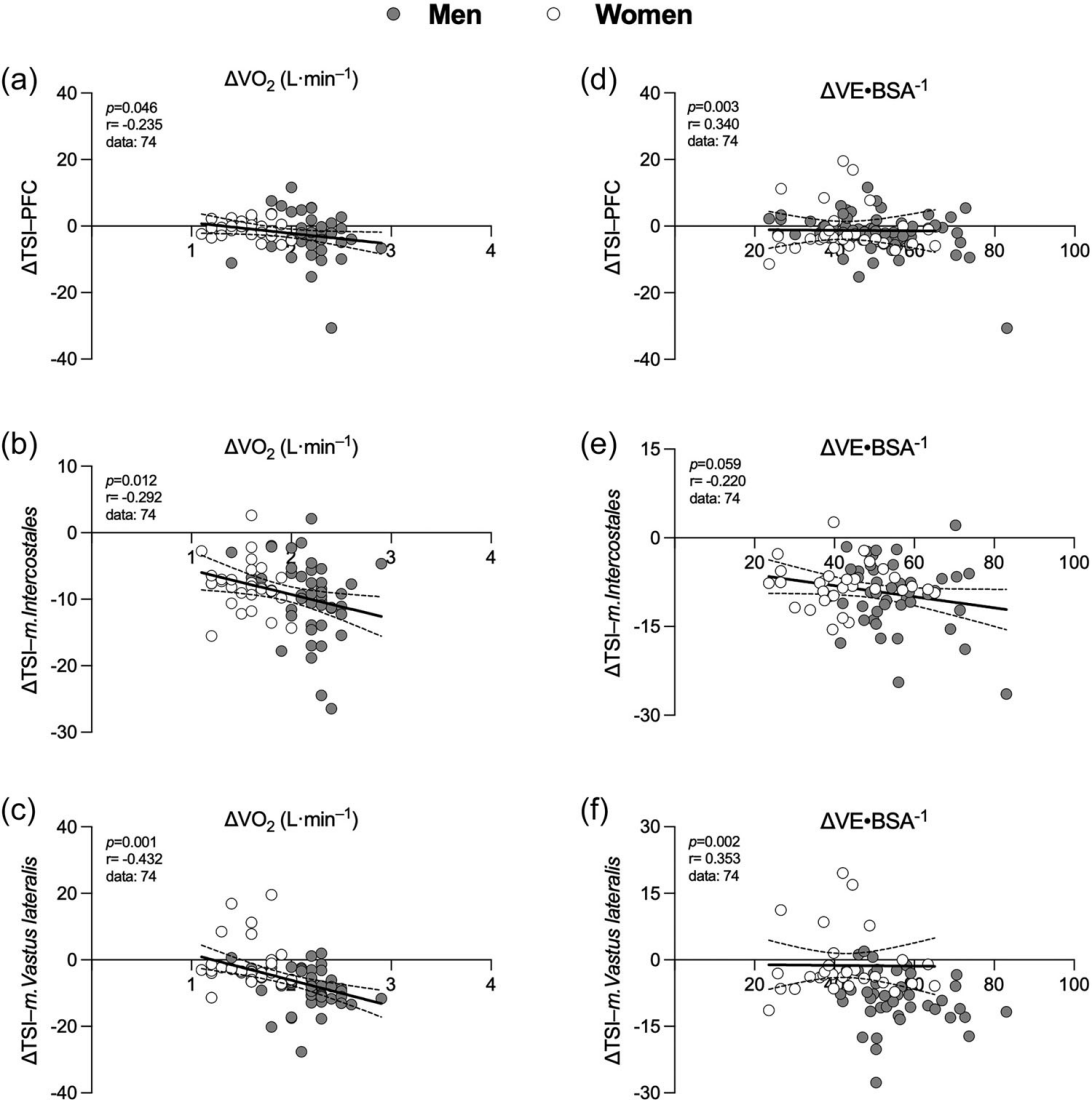
Associations between the changes (Δ) of near‐infrared spectroscopy data (TSI) at the prefrontal cortex (PFC), m. intercostales and m. vastus lateralis with $\Delta {\dot{V}}_{O_{2}}$ and ΔV˙E/ΔV˙EBSABSA (*n* = 74): (a) ΔTSI at PFC with $\Delta {\dot{V}}_{O_{2}}$; (b) ΔTSI at m. intercostales with $\Delta {\dot{V}}_{O_{2}}$; (c) ΔTSI at m. vastus lateralis with $\Delta {\dot{V}}_{O_{2}}$; (d) ΔTSI at PFC with ΔV˙E/ΔV˙EBSABSA; (e) ΔTSI at m. intercostales with ΔV˙E/ΔV˙EBSABSA; and (f) ΔTSI at m. vastus lateralis with ΔV˙E/ΔV˙EBSABSA. Abbreviations: TSI, tissue saturation index; V˙E/V˙EBSABSA, the ratio between lung ventilation (${\dot{V}}_{E}$) and body surface area (BSA); ${\dot{V}}_{O_{2}}$ , oxygen uptake.

## 4 DISCUSSION

In this study, we investigated sex differences in brain, respiratory and locomotor muscle oxygenation during incremental exercise to exhaustion in individuals trained in endurance sports. The results revealed distinct oxygenation patterns between men and women across the three tissues assessed. Men showed greater increases in oxygen delivery markers (ΔO_2_‐Hb and ΔT‐Hb) in the PFC and higher oxygen extraction (ΔH‐Hb) in the locomotor muscles (m. vastus lateralis). In contrast, women exhibited greater oxygen extraction in respiratory muscles (m. intercostales) accompanied by lower oxygen delivery markers in this tissue. These sex‐specific patterns in tissue oxygenation were observed despite both groups exercising to volitional exhaustion and meeting established criteria for maximal effort.

### 4.1 Sex differences in PFC oxygenation

Men exhibited a greater increase in ΔO_2_‐Hb and ΔT‐Hb, along with a more pronounced decline in ΔTSI, whereas no significant sex differences were observed in ΔH‐Hb, consistent with previous findings (Orcioli‐Silva et al., 2024). This pattern suggests that men might experience greater oxygen delivery to the PFC during exercise. However, it is important to acknowledge that the observed increase in ΔO_2_‐Hb and ΔT‐Hb might reflect increased skin blood flow in the forehead region for thermoregulatory purposes, in addition to cerebral perfusion. NIRS signals from frontal placements can include substantial contributions (20%–30%) from extracerebral vasculature, which makes it difficult to attribute changes definitively to cerebral haemodynamics alone (Takahashi et al., 2011).

The increase in ΔO_2_‐Hb observed in male subjects until exhaustion, despite hyperventilation‐induced hypocapnia that would typically cause cerebral vasoconstriction, presents an apparent physiological contradiction. Several explanations might account for this finding: (1) the magnitude of hypocapnia might not have been sufficient to overcome other vasodilatory stimuli, such as increased metabolic demand or sympathetic activation; (2) young healthy males might exhibit robust cerebral autoregulation, which maintains perfusion despite moderate CO_2_ reductions; or (3) the NIRS signal might have been influenced by increased cutaneous blood flow, which obscures potential reductions in cerebral perfusion. Future studies should consider taking simultaneous measurements of skin blood flow (e.g., using laser Doppler flowmetry) to distinguish cerebral contributions better from extracerebral contributions to the NIRS signal.

In contrast, female subjects showed a more subtle increase in ΔO_2_‐Hb and ΔT‐Hb during incremental exercise, suggesting a potentially different pattern of oxygen delivery to the PFC compared with males. This pattern might be related to cerebral vasoconstriction driven by hyperventilation‐induced hypocapnia, as suggested by the trend towards higher V˙E/V˙EV˙CO2V˙CO2 and lower $P_{\mathrm{ET},CO_{2}}$ in women up to 90% ${\dot{V}}_{O_{2}\mathrm{peak}}$. Although this trend was not statistically significant, it aligns with previous findings indicating that women exhibit a greater ventilatory response to exercise (Dominelli et al., 2015; Espinosa‐Ramírez et al., 2021; Kipp et al., 2024), which might lead to a more pronounced drop in the arterial partial pressure of carbon dioxide and reduced cerebral perfusion (Ashley et al., 2020; Querido & Sheel, 2007; Smith & Ainslie, 2017).

Notably, despite the reduced oxygen delivery, female subjects exhibited greater ΔTSI values than males at moderate to high intensities, suggesting that factors beyond oxygen delivery and extraction might regulate oxygen uptake in the PFC during exercise. One possible explanation lies in sex differences in basal cerebral blood flow. Across all ages, women exhibit ∼11% higher cerebral blood flow than men (Rodriguez et al., 1988), a difference that can reach 15% in young adults (Gur et al., 1982). This elevated cerebral blood flow in women has been attributed, in part, to higher oestrogen levels, which are associated with increased prostaglandin concentrations, a vasoactive compound thought to enhance the vasodilatory response to CO_2_ and play a key role in the regulation of cerebral blood flow (Kastrup et al., 1999). In addition, young women show more effective cerebral autoregulation, regardless of menstrual cycle phase, indicating a superior ability to maintain stable oxygen and nutrient delivery despite fluctuations in blood pressure (Favre & Serrador, 2019). Consequently, the higher ΔTSI values observed in women might reflect compensatory mechanisms that mitigate the impact of lower oxygen delivery at high intensities compared with men. However, despite these advantages, the limited oxygen availability to the PFC suggests that women might remain more susceptible to central mechanisms of exercise limitation.

Another factor that might have influenced NIRS responses in the PFC is the difference in aerobic fitness levels or ${\dot{V}}_{O_{2}\mathrm{peak}}$, which were higher in men. Previous studies have shown that individuals with greater aerobic capacity can experience a more pronounced cerebral demand in response to moderate‐intensity exercise (Li et al., 2024). Given that no acute brain responses at multiple intensities across fitness levels were assessed, it is not possible to account fully for how aerobic fitness might have modulated the observed sex differences in brain oxygenation.

The potential effect of age on these sex differences in PFC responses also warrants consideration. Some studies suggest that older women with higher aerobic fitness might have better cerebral oxygenation (Orcioli‐Silva et al., 2024; Smith & Ainslie, 2017), whereas others have found no sex differences in respiratory muscle oxygen consumption in older adults (Kipp et al., 2024). This highlights the importance of considering age and fitness status when interpreting haemodynamic changes induced by exercise across different tissues. Future studies should investigate more thoroughly the interaction between aerobic capacity, age and sex in shaping brain oxygenation responses to exercise.

### 4.2 Oxygenation responses in respiratory muscles

In the m. intercostales, significant sex‐by‐intensity interactions were found for both ΔO_2_‐Hb and ΔT‐Hb, with female subjects showing lower values than males across these variables. Notably, female subjects demonstrated an immediate decline in ΔO_2_‐Hb from baseline during the warm‐up phase and maintained significantly lower levels throughout all intensities. They also experienced greater declines in ΔT‐Hb at moderate to high intensities (≥50% ${\dot{V}}_{O_{2}\mathrm{peak}}$). In contrast, ΔH‐Hb was significantly higher in female subjects from warm‐up to 90% of ${\dot{V}}_{O_{2}\mathrm{peak}}$, suggesting a greater degree of oxygen extraction in response to rising ventilatory demands.

These findings imply that women might experience a greater cost of breathing at moderate to high exercise intensities, characterized by more pronounced respiratory muscle deoxygenation and reduced blood volume. This is likely to be a reflection of increased metabolic demand, consistent with previous research (Dominelli et al., 2015; Kipp et al., 2024). However, the absence of significant sex differences in ΔTSI suggests that women might rely on compensatory mechanisms to maintain tissue saturation despite lower oxygen availability. This supports the notion that oxygen uptake is regulated by factors beyond delivery and extraction alone.

In terms of ventilatory responses, female subjects exhibited lower V˙E/V˙EBSABSA despite having similar respiratory rates to males, indicating reduced ventilatory efficiency and a higher metabolic burden on the respiratory muscles. These findings align with previous studies showing that, for a given level of ventilation, women experience a higher work of breathing and greater oxygen consumption in the respiratory muscles (Espinosa‐Ramírez et al., 2021; Santisteban et al., 2022; Sheel et al., 2016). Anatomically, women tend to have smaller lungs, narrower airways and reduced lung compliance, leading to lower lung volumes and higher airway resistance (Sheel et al., 2016). Additionally, their smaller ribcages and reduced diaphragm excursion contribute to a greater reliance on accessory respiratory muscles during exercise (LoMauro & Aliverti, 2018), predisposing women to earlier onset of respiratory muscle fatigue and further increasing the metabolic cost of breathing during high‐intensity exercise.

As exercise intensity increases, CO_2_ production rises as a byproduct of increased metabolic activity, as reflected in higher ${\dot{V}}_{CO_{2}}$ values for both male and female subjects during CPET (Skinner & McLellan, 1980). Approaching VT2, increased [H^+^] triggers hyperventilation, further amplified by the metaboreflex, which increases respiratory muscle activation to meet rising metabolic demands. Owing to reduced ventilatory efficiency, women appear to rely more heavily on accessory respiratory muscles, evidenced by greater ΔH‐Hb levels and lower V˙E/V˙EBSABSA. This might contribute to central limiting mechanisms in women, whereby increased respiratory muscle workload and metabolic strain influence cerebral regions involved in motor task planning, as previously discussed.

### 4.3 Oxygenation in the locomotor muscles

In the m. vastus lateralis, male subjects exhibited increases in ΔH‐Hb and ΔT‐Hb from baseline, whereas females showed decreases in these variables. This pattern suggests an enhanced local blood volume, as evidenced by the ΔT‐Hb increase, and a greater reliance on oxygen extraction, as reflected by the rise in ΔH‐Hb, supporting the idea of a more prominent peripheral contribution to exercise performance, which has been reported previously (Espinosa‐Ramírez et al., 2021). Furthermore, men generally achieve higher workloads during maximal tests, probably attributable to larger muscle cross‐sectional areas, longer limb lengths and higher testosterone levels (Hunter et al., 2023). In line with this, male subjects in our study reached significantly higher absolute and body weight‐adjusted workloads than females, further emphasizing the role of peripheral factors in sustaining physical performance.

The immediate and sustained decline in ΔTSI observed in male subjects from baseline to exhaustion supports this conclusion. Additionally, male subjects exhibited significantly lower ΔTSI values than females across all intensities of CPET, consistent with previous findings (Espinosa‐Ramírez et al., 2021). This pattern highlights a higher peripheral metabolic cost in men, which might contribute to peripheral limitations to performance. The lack of sex differences in ΔO_2_‐Hb reinforces this interpretation, because both sexes exhibited similar trends in muscle oxygenation. However, despite this similarity in oxygenation, the greater peripheral contributions to exercise performance in male subjects are reflected in the more pronounced declines in ΔTSI and the higher workloads achieved, underscoring the importance of peripheral adaptations in the performance capacity of males.

One potential mechanism underlying these sex differences involves disparities in muscle mass and fibre composition. Men generally possess greater muscle mass and a higher proportion of fast‐twitch muscle fibres. In the m. vastus lateralis, men typically have ∼64% fast‐twitch fibres, compared with 56% in women (Staron et al., 2000). These fibres exhibit greater glycolytic activity and are recruited preferentially at higher intensities, promoting reliance on anaerobic glycolysis. This metabolic shift leads to the accumulation of byproducts such as H^+^, which reduce the affinity of haemoglobin for oxygen. This mechanism might explain the higher ΔH‐Hb and lower ΔTSI values observed in male subjects, indicating greater deoxygenation in locomotor muscles under intense workloads.

In addition to muscle composition, other physiological factors contribute to these differences. Men typically exhibit higher cardiac output and lower peripheral resistance in active leg muscles during exercise, enhancing muscle perfusion compared with women (Hunter et al., 2023; Leahy et al., 2023; Santisteban et al., 2022; Smith & Ainslie, 2017). Hormonal influences, particularly testosterone, can also facilitate increased muscle blood flow in men. Together, these structural, functional and hormonal differences are likely to explain the elevated peripheral metabolic demand observed in men during high‐intensity exercise and their greater susceptibility to peripheral factors that might ultimately limit performance.

### 4.4 Limitations

This study has limitations that should be acknowledged. First, this study used continuous‐wave NIRS technology, which assumes a constant differential pathlength factor. This assumption limits the ability to measure the absolute concentration of chromophores. Consequently, continuous‐wave NIRS can detect only relative changes in haemodynamic variables from an arbitrary baseline, rather than providing absolute quantification of tissue oxygenation or haemoglobin concentration. Therefore, our findings reflect patterns of change rather than the absolute state of tissue oxygenation. Comparisons between sexes represent differences in oxygenation dynamics rather than definitive assessments of the absolute extent of fatigue or physiological exhaustion. Future research should consider using NIRS systems with time‐resolved or time‐domain spectroscopy, which enable the measurement of absolute concentrations during exercise (e.g., NIRBOX™ from PIONIRS, Milan, Italy).

Second, the NIRS measurements from the forehead region are susceptible to contamination from extracerebral tissues, especially skin blood flow. During high‐intensity exercise, cutaneous vasodilatation for thermoregulation increases superficial blood flow, which can contribute significantly to the NIRS signal. Our observation of increased ΔO_2_‐Hb in men despite hyperventilation‐induced hypocapnia might partly reflect increased skin perfusion rather than cerebral haemodynamics exclusively. This limitation is inherent to frontal NIRS placements and cannot be resolved fully without complementary measurements, such as laser Doppler flowmetry to measure skin blood flow or more sophisticated NIRS systems with better depth resolution. Future studies should incorporate these methodologies to distinguish cerebral from extracerebral contributions to the observed signals better.

Third, given that male subjects began CPET at higher absolute workloads (100 W, vs. 80 W for females) and progressed at larger increments (25 vs. 20 W min^−1^), it is difficult to determine whether the observed differences in deoxygenation, particularly in locomotor muscles, are attributable solely to sex‐based physiological differences or in partly to the protocol design. Although we analysed the data as a percentage of ${\dot{V}}_{O_{2}\mathrm{peak}}$ to normalize the relative intensity, the higher absolute mechanical workload experienced by male subjects throughout the protocol might have contributed to their greater peripheral deoxygenation. This confounding factor is difficult to avoid when comparing sexes, owing to inherent differences in absolute exercise capacity. However, it should be considered when interpreting our findings, especially those regarding the m. vastus lateralis.

Fourth, although female subjects were tested in the luteal phase based on self‐reported menstrual cycle tracking, there was no hormonal confirmation (e.g., serum progesterone or oestradiol measurements). Although young women have shown effective mechanisms of cerebral autoregulation during exercise, independent of menstrual cycle phase and fluctuations in blood pressure, hormonal variations should nonetheless be considered to characterize better how menstrual cycle‐related changes in cardiovascular and cerebrovascular function, including cerebral blood flow and autoregulation, influence exercise performance. The absence of biochemical verification and the absence of analysis of variability across different phases of the menstrual cycle limit the generalizability of our findings to all phases. Future studies should include hormonal confirmation and, ideally, test female subjects across multiple phases of the cycle to understand better how hormonal status modulates exercise‐induced oxygenation responses.

Fifth, this study did not include continuous arterial pressure monitoring or cardiac output measurements during CPET. The assessment of arterial blood pressure, particularly mean arterial pressure, is crucial for a more comprehensive understanding of cerebral perfusion dynamics, especially in high‐intensity conditions, in which autoregulatory mechanisms might be challenged. Incorporation of mean arterial pressure measurements in future research is strongly recommended to contextualize better the cerebral oxygenation responses and their role in exercise tolerance. Additionally, the characterization of cardiovascular variables, such as cardiac output and peripheral vascular resistance, can help us to quantify the changes induced by exercise in blood flow at the muscle level. Despite these limitations, the present findings offer meaningful contributions to the understanding of sex‐based differences in oxygenation dynamics during exercise.

Sixth, the correlational analyses presented in Figure 5 pooled data across all exercise intensities and participants. Although this approach reveals overall associations between tissue oxygenation and metabolic/ventilatory demands, it does not account for the non‐independence of repeated measures within individuals. Future studies should use mixed‐effects models to handle the hierarchical structure of such data in a more appropriate manner.

### 4.5 Implications for exercise limitation and training strategies

The present findings suggest that men and women exhibit distinct patterns of tissue oxygenation during exercise, which might reflect different physiological constraints on performance. In male subjects, the observed increases in PFC oxygenation markers, coupled with greater oxygen extraction in the m. vastus lateralis, suggest that exercise cessation might be driven by peripheral factors. Men appear able to maintain relatively stable cerebral oxygenation patterns while demonstrating substantial oxygen extraction in the locomotor muscles. In contrast, female subjects exhibited greater deoxygenation in the m. intercostales and demonstrated patterns that suggest potential limitations in cerebral oxygen delivery. This indicates that central mechanisms might play a significant role in performance constraints.

### 4.6 Evidence‐based training strategies

Owing to these distinct oxygenation patterns, different training interventions are required for each sex. For women, strategies that improve respiratory muscle endurance, such as specific respiratory muscle training, have been shown to reduce the work of breathing. These strategies might also reduce respiratory‐related limitations (HajGhanbari et al., 2013). Additionally, interventions that improve ventilatory efficiency could theoretically reduce hyperventilation‐induced cerebral vasoconstriction. However, further research is needed to investigate this application. Training approaches targeting peripheral adaptations, such as high‐intensity interval training to enhance mitochondrial capacity and oxygen utilization in locomotor muscles or blood flow restriction training to promote peripheral vascular adaptation, might be particularly beneficial for men owing to their greater peripheral oxygen extraction pattern.

### 4.7 Proposed directions for future research

This study provides valuable findings for future research, but they need to be confirmed through controlled studies. Training in conditions of varying oxygen availability (e.g., intermittent hypoxic training or hyperoxic training) might differentially affect central versus peripheral limitations and could be explored as sex‐specific interventions (Kowalski et al., 2025). Additionally, cognitive training or neurofeedback has the potential to enhance cerebral efficiency during exercise, which is an unexplored area that could be particularly relevant for addressing central limitations in women. Finally, interventions that combine respiratory muscle function and cerebrovascular reactivity through optimization of the breathing pattern require systematic investigation.

## 5 CONCLUSION

In conclusion, this study highlights the importance of incorporating sex‐specific physiological differences when assessing exercise performance and developing training strategies. The findings support the hypothesis that women might be more susceptible to central patterns of oxygenation, suggesting limitations in cognitive motor control regions, whereas men might be more prone to peripheral limiting factors. These findings emphasize the need for tailored training strategies to address the distinct physiological constraints experienced by each sex during exercise, particularly across varying exercise intensities.

## AUTHOR CONTRIBUTIONS

Felipe Contreras‐Briceño designed the study. Daniel Ramos‐López, Benjamín Díaz‐Ortiz, Cristóbal Baumann‐Biancani, Kamilo Hunger‐Abbott, Matías Herrera‐Matas, Felipe Contreras‐Briceño and Maximiliano Espinosa‐Ramírez organized and performed experiments. Raúl Caulier‐Cisterna, Andrés Vega‐Moraga, Luigi Gabrielli‐Nervi, Hugo E. Verdejo and Karol Ramírez‐Parada analysed and interpreted the data. Felipe Contreras‐Briceño, Daniel Ramos‐López, Vitor A. Lira, Maximiliano Espinosa‐Ramírez and Karol Ramírez‐Parada drafted and edited the manuscript. All authors approved of the final version of the manuscript and agree to be accountable for all aspects of the work in ensuring that questions related to the accuracy or integrity of any part of the work are appropriately investigated and resolved. All persons designated as authors qualify for authorship, and all those who qualify for authorship are listed. The content is solely the responsibility of the authors and does not necessarily represent the official views of Pontificia Universidad Católica de Chile (Chile), Universidad Tecnológica Metropolitana (Chile), University of Iowa (USA), Universidad Santo Tomás (Chile) and the American College of Sports Medicine (ACSM, USA).

## CONFLICT OF INTEREST

None declared.

## Supporting information




**Figure S1**. STROBE guidelines flow diagram of participants.

## Data Availability

Source data for this study are not publicly available owing to privacy or ethical restrictions. The source data are available to verified researchers upon request by contacting the corresponding author.
