# Supplementary material for: Sex differences in the prefrontal cortex and muscle oxygenation during exercise until exhaustion in endurance‐trained individuals
Source: Exp Physiol. 2025 Nov 29;111(11):5136–53. doi: 10.1113/EP093287 (PMC13394771; doi:10.1113/EP093287)
Supplement: Supplementary file 1 [file EPH-111-5136-s001.pdf]

Supplementary Material

S1. Participant Flow Diagram.

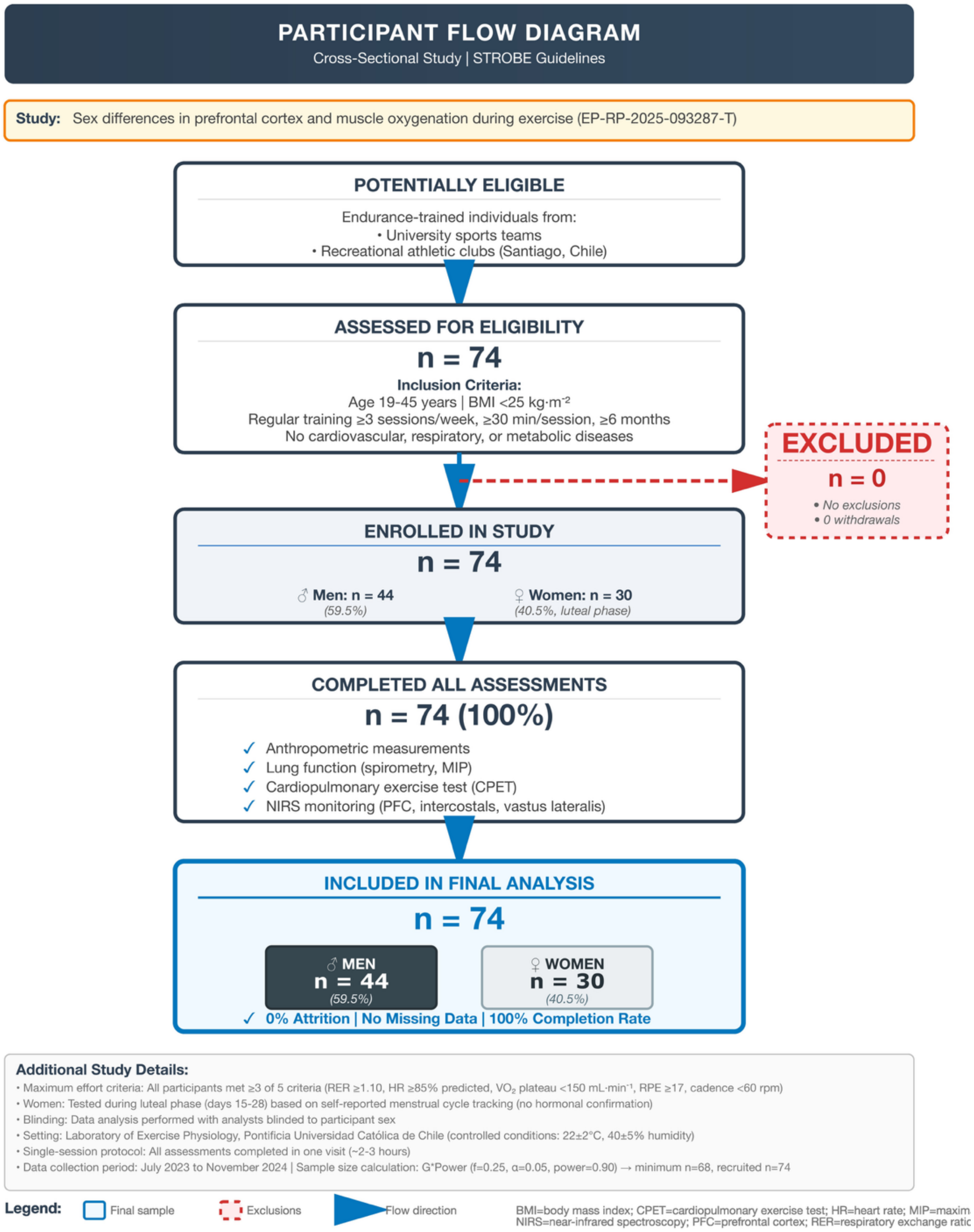

Flow of participants through the study from assessment for eligibility through final analysis. All 74 individuals assessed for eligibility were enrolled and completed all study procedures. There were no exclusions, withdrawals, or missing data. Men comprised 59.5% (n=44) and women 40.5% (n=30) of the final sample. All participants met established criteria for maximum effort during cardiopulmonary exercise testing (CPET); NIRS, Near–Infrared Spectroscopy.
